# Hyperglycemia-induced diaphragm weakness is mediated by oxidative stress

**DOI:** 10.1186/cc13855

**Published:** 2014-05-03

**Authors:** Leigh A Callahan, Gerald S Supinski

**Affiliations:** 1 Department of Internal Medicine, Division of Pulmonary, Critical Care and Sleep Medicine, University of Kentucky, 740 South Limestone Room L-543, Lexington, KY 40536-0284, USA

## Abstract

**Introduction:**

A major consequence of ICU-acquired weakness (ICUAW) is diaphragm weakness, which prolongs the duration of mechanical ventilation. Hyperglycemia (HG) is a risk factor for ICUAW. However, the mechanisms underlying HG-induced respiratory muscle weakness are not known. Excessive reactive oxygen species (ROS) injure multiple tissues during HG, but only one study suggests that excessive ROS generation may be linked to HG-induced diaphragm weakness. We hypothesized that HG-induced diaphragm dysfunction is mediated by excessive superoxide generation and that administration of a specific superoxide scavenger, polyethylene glycol superoxide dismutase (PEG-SOD), would ameliorate these effects.

**Methods:**

HG was induced in rats using streptozotocin (60 mg/kg intravenously) and the following groups assessed at two weeks: controls, HG, HG + PEG-SOD (2,000U/kg/d intraperitoneally for seven days), and HG + denatured (dn)PEG-SOD (2000U/kg/d intraperitoneally for seven days). PEG-SOD and dnPEG-SOD were administered on day 8, we measured diaphragm specific force generation in muscle strips, force-pCa relationships in single permeabilized fibers, contractile protein content and indices of oxidative stress.

**Results:**

HG reduced diaphragm specific force generation, altered single fiber force-pCa relationships, depleted troponin T, and increased oxidative stress. PEG-SOD prevented HG-induced reductions in diaphragm specific force generation (for example 80 Hz force was 26.4 ± 0.9, 15.4 ± 0.9, 24.0 ± 1.5 and 14.9 ± 0.9 N/cm^2^ for control, HG, HG + PEG-SOD, and HG + dnPEG-SOD groups, respectively, *P* <0.001). PEG-SOD also restored HG-induced reductions in diaphragm single fiber force generation (for example, Fmax was 182.9 ± 1.8, 85.7 ± 2.0, 148.6 ± 2.4 and 90.9 ± 1.5 kPa in control, HG, HG + PEG-SOD, and HG + dnPEG-SOD groups, respectively, *P* <0.001). HG-induced troponin T depletion, protein nitrotyrosine formation, and carbonyl modifications were largely prevented by PEG-SOD.

**Conclusions:**

HG-induced reductions in diaphragm force generation occur largely at the level of the contractile proteins, are associated with depletion of troponin T and increased indices of oxidative stress, findings not previously reported. Importantly, administration of PEG-SOD largely ablated these derangements, indicating that superoxide generation plays a major role in hyperglycemia-induced diaphragm dysfunction. This new mechanistic information could explain how HG alters diaphragm function during critical illness.

## Introduction

A major consequence of ICU acquired skeletal muscle weakness (ICUAW) is respiratory muscle dysfunction which leads to prolonged duration of mechanical ventilation [1]. Importantly, a number of recent studies indicate that critically ill mechanically ventilated patients have severe respiratory muscle weakness, with diaphragm force generation averaging only 23% of the level observed in healthy individuals [2-5]. In addition, clinical studies have identified a number of factors that are thought to contribute to ICU acquired diaphragm weakness including mechanical ventilation induced respiratory muscle inactivity, sepsis/infections, use of corticosteroids and hyperglycemia [6-8].

Several recent reports indicate that hyperglycemia detrimentally affects respiratory muscle performance in ICU patients [9-13]. Specifically, Van den Berghe *et al*. found that intensive insulin therapy (that is, administration of insulin to maintain blood glucose in the 80 to 110 mg/dl range) in ICU patients markedly reduced the time required to wean patients from mechanical ventilation, shortened ICU stay, and was associated with decreased incidence of ICUAW [9,13]. In later work, this group showed that electromyographic abnormalities consistent with the diagnosis of ICUAW were prevented with intensive control of blood glucose levels [10,12]. In addition, more strict control of glucose reduced the duration of mechanical ventilation [10]. Moreover, numerous clinical studies in patients with a variety of diagnoses have now established that the presence of acute hyperglycemia is associated with poor outcomes, including increased mortality during critical illness [14,15].

The strongest evidence that hyperglycemia is a risk factor for acquired respiratory muscle weakness in ICU patients is borne out by these clinical observations [16]. However, the cellular mechanisms by which acute hyperglycemia induces respiratory muscle weakness are not known. On the other hand, previous studies indicate that hyperglycemia induces excessive generation of superoxide and other reactive oxygen species (ROS) in several tissues, including the vascular endothelium, the retina and kidneys [17-19] and that excessive ROS generation mediates the long term complications of diabetes in these organs. In addition, excessive ROS formation has been shown to mediate the development of skeletal muscle contractile dysfunction in several diseases, including sepsis, heart failure and inactivity induced muscle atrophy [20-26].

Only one previous animal study has provided a potential link between hyperglycemia induced diaphragm weakness and excessive ROS generation. In this study, Hida *et al*. [27] found that after two weeks of streptozotocin (STZ) induced hyperglycemia, diaphragm specific force generation was significantly reduced and that administration of a non-specific antioxidant, N-acetyl cysteine (NAC) restored these reductions. In their conclusion, these authors speculated that NAC may have exerted its effects either by scavenging free radicals or by inhibiting tumor necrosis factor [27]. However, this study did not determine how hyperglycemia altered diaphragm contractility or assess if hyperglycemia actually induced oxidative stress in the diaphragm.

Given the recent recognition that hyperglycemia is a major clinical risk factor for ICU acquired diaphragm weakness, that excessive ROS generation plays a role in hyperglycemia-induced organ injury in multiple tissues (and, possibly, the diaphragm based on the single study cited above) and that excessive ROS generation has been implicated in diaphragm weakness in other conditions, we postulated that hyperglycemia may induce excessive superoxide generation in the diaphragm and may be responsible for the development of hyperglycemia-induced reductions in diaphragm contractility. Therefore, the purpose of the present study was to test this hypothesis using an animal model of hyperglycemia (that is, STZ-induced hyperglycemia) and to determine further the specific role of oxidative stress in modulating diaphragm dysfunction during hyperglycemia. We compared diaphragm force generation in intact diaphragm muscle strips, single fiber force-pCa relationships, levels of key contractile proteins and indices of ROS-mediated protein modifications in four groups of rats including sham treated euglycemic controls, hyperglycemic animals, hyperglycemic animals given a specific superoxide scavenger, polyethylene glycol-superoxide dismutase (PEG-SOD) and hyperglycemic animals given enzymatically inactive, denatured PEG-SOD. Data were analyzed to determine the subcellular targets by which hyperglycemia reduces diaphragm force generating capacity and to determine if treatment with active PEG-SOD, a specific scavenger of excessive superoxide generation, would reverse hyperglycemia-induced alterations in the diaphragm.

We chose to focus on the diaphragm specifically because respiratory muscle weakness in the ICU is a major clinical problem [2,3], and diaphragm weakness in ICU patients has been shown to result in poor outcomes, including prolonged duration of mechanical ventilation, increased incidence of patient transfers to long term ventilator units and higher ICU mortality [2,3]. In addition, there is strong clinical data showing that strict glucose control reduces ICU acquired diaphragm weakness, shortening duration of mechanical ventilation and ICU length of stay in critically ill patients [9,10,16]. Finally, there is a paucity of mechanistic information regarding the effects of hyperglycemia on the diaphragm.

## Materials and Methods

All studies were approved by the University of Kentucky Institutional Animal Care and Use Committee (Animal assurance number A3336-01). Care and handling of animals was in accordance with the guidelines of the National Institutes of Health Office of Lab Animal Welfare.

### Experimental models and protocols

Experiments were performed using adult male Sprague Dawley rats weighing between 250 and 350 gm. Animals had unrestrained access to food and water throughout the study. To prevent dehydration, animals were housed individually and supplied with two water sources. Animals were monitored throughout experiments by members of the University of Kentucky Division of Laboratory Animal Resources as well as research personnel.

In pilot studies, we assessed diaphragm contractile function at several time points and noted consistent reductions in diaphragm contractility at two weeks. As a result, we chose to perform studies at this time point. This time point is relevant to ICU acquired diaphragm weakness which is most often clinically recognized when patients are unable to be weaned, which usually occurs after at least seven days of mechanical ventilation [6,8].

Four groups of animals were studied, including: (1) sham treated euglycemic controls, (2) streptozotocin (STZ)-treated hyperglycemic animals, (3) STZ-treated hyperglycemic animals given a superoxide scavenger (PEG-SOD), and (4) STZ-treated hyperglycemic animals given enzymatically inactive, denatured PEG-SOD. Hyperglycemia was induced by a single tail vein injection of STZ (60 mg/kg) (Sigma Chemicals, St Louis, MO, USA) dissolved in citrate buffer (pH 4.5). Sham-treated controls were injected via tail vein with an equivalent volume of citrate buffer alone. Tail vein blood was used to assess blood glucose levels on days 2 and 7 post injection of STZ or buffer in all animals to document hyperglycemia (glucose >300 mg/dl) in STZ-treated animals and euglycemia in controls. Measurements were performed using a commercially available glucometer (Precision Xtra, Abbott, Alameda, CA, USA). At one week after the initial injections of STZ or citrate buffer, animals were then treated with intraperitoneal injections of either saline, PEG-SOD (2,000 units/kg/day), or denatured PEG-SOD (heat denatured 2,000 units/kg/day) for a total of seven days. On the day of sacrifice (two weeks after initial injections), a final blood glucose determination was made prior to administration of anesthetics, and animals were subsequently deeply anesthetized (pentobarbital 150 mg/kg injected intraperitoneally) and diaphragms harvested. A portion of the left costal diaphragm was used immediately to assess force frequency relationships, a second section was removed for determination of single fiber force-pCa relationships, and a third section was frozen and stored (at -80°C) for biochemical assessments of contractile protein levels and ROS-mediated protein modifications.

### Measurement of diaphragm force-frequency relationships

Diaphragm specific force generation was assessed as previously reported [28-30]. Intact diaphragm strips were dissected from the left costal diaphragm and mounted vertically in water-jacketed organ baths (Radnoti, Monrovia, CA, USA) containing Krebs-Henseleit solution (25°C, curare 50 mg/L, pH 7.40, NaCl 135 mM, KCl 5 mM, dextrose 11.1 mM, CaCl_2_ 2.5 mM, MgSO_4_ 1 mM, NaHCO_3_ 14.9 mM, NaHPO_4_ 1 mM, insulin 50 units/L, 95% O_2_/5% CO_2_). The rib end of the strips was attached to the bottom of the baths by silk ties, and the tendon end was tied to a force transducer (Grass Technologies, West Warwick, RI, USA). Platinum field electrodes were placed around strips and connected to an amplifier (Biomedical Technology of America, Cleveland, Ohio, USA) attached to a Grass S48 stimulator (Grass Technologies). After a 15 minute equilibrium period, muscle length was adjusted to L_o_ (the length at which force generation was maximum), stimulation current was adjusted to supramaximal levels and a force-frequency curve was constructed by stimulating strips at 1, 10, 20, 50, and 80 Hz (train duration 800 msec) with a 30 second rest period between adjacent stimulus trains. Force was recorded with a Gould 2600 strip chart recorder (Gould Instruments System, Cleveland, Ohio, USA). After force measurements were completed, transducers were calibrated with standard weights. Cross sectional area was calculated as muscle strip weight divided by muscle density (1.06) and muscle length; diaphragm specific force generation was then calculated as raw force divided by cross sectional area [31].

### Assessment of force-pCa relationships in single permeabilized diaphragm fibers

Force-pCa curves from single permeabilized diaphragm fibers were determined as previously reported [24,32-34]. At the time of animal sacrifice, a portion of the diaphragm was carefully removed from its intercostal insertions and placed in a dissecting dish with a relaxing solution containing (in mM): 1.0 Mg^2+^, 5.0 MgATP, 15 phosphocreatine, 140.0 potassium methanesulfonate, 50.0 imidazole and 10.0 ethylene glycol tetraacetic acid (EGTA), with pCa >8.5, pH 7.0 and an ionic strength of 200. Protease inhibitors were added to the solution to protect the fibers from the damaging effects of proteolysis and included the following: 0.1 mM phenylmethysulfonyl fluoride, 0.1 mM leupeptin, 1.0 mM benzamidine and 10 μM aprotinin. The diaphragm was then divided into small strips and stored at -20°C in the relaxing solution containing 50% glycerol and protease inhibitors for later experimentation. For the storage solution, CTP was used instead of ATP to prevent phosphorylation of the myosin light chains. All single-fiber assessments were completed within one week of animal sacrifice.

On the day that single fiber characteristics were assessed, diaphragm strips were removed from the storage solution, placed in the relaxing solution and equilibrated to room temperature. Bundles of approximately 10 fibers were gently separated from mid-costal diaphragm strips by pulling on one end of the muscle with a pair of fine-tipped forceps while the other end of the muscle was stationary. Following this, fiber bundles were permeabilized for 30 minutes in the relaxing solution containing 0.1% Triton X-100, an ionic detergent that eliminates the membranes of the sarcolemma, sarcoplasmic reticulum and mitochondria, leaving only the contractile proteins intact.

After incubation, bundles were removed from Triton X-100, placed in the relaxing solution, and individual fibers teased from the muscle bundles. Single fibers were then mounted between an optoelectric force transducer (Scientific Instruments, Heidelberg, Germany) and a movable arm by wrapping the ends of each fiber around stainless steel clips. Fibers were adjusted to achieve a resting sarcomere length of 2.6 μm as indicated by its helium-neon laser diffraction pattern. Length was constant throughout the protocol. The cross-sectional area of each fiber was determined after adjustment of the sarcomere length by measuring the diameter of the fiber using a micrometer attached to the eyepiece of the microscope and area was subsequently calculated assuming a cylindrical shape for the fiber.

Force versus pCa curves were then constructed for fibers by immersing them in solutions of increasing calcium concentrations and recording tension on a strip recorder. Once peak tension was achieved in a given pCa solution, fibers were rapidly switched to the next solution by means of a spring-loaded Plexiglas tray. The composition of all solutions for this study was calculated by using a computer program (Borland International, Scotts Valley, CA, USA) that takes into account stability constants and stock solutions to produce final solutions of the correct ionic strength and pCa (12). Specifically, the pCa solutions contained (in mM): 1.0 Mg^2+^, 1.0 MgATP, 15 phosphocreatine, 110.0 potassium methanesulfonate, 20.0 imidazole and 5.0 EGTA, with pH 7.0, and ionic strength of 200. Addition of different amounts of calcium yielded solutions of the desired pCa. To establish the force versus pCa relationship, fibers were submerged in a solution containing no added calcium (pCa 8.5), followed by sequential exposure to 13 different calcium solutions, namely pCa 6.0, 5.90, 5.80, 5.75, 5.70, 5.65, 5.60, 5.55, 5.50, 5.40, 5.30, 5.20 and 5.0. These solutions correspond to a range of calcium concentrations of 10^-6^ to 10^-5^ M. Data were assessed using SigmaPlot software (version 12.0, Jandel Scientific) to determine the constant *N* related to the steepness of the force versus pCa relationship (*N* is a measure of the extent of cooperativity among the thin filaments) and the calcium concentration required for half-maximal activation (Ca_50_) (K) values for the force-pCa relationships from a best fit of the data to the modified Hill equation: % maximum force = 100[Ca^2+^]^N^/((K)^N^ + [Ca^2+^]^N^). Averages and standard errors of the mean for *N* values, Ca_50_, cross-sectional area, percentage of F_max_ and absolute force (normalized for cross-sectional area) for individual diaphragm fibers were calculated for fibers from the experimental groups.

### Determination of diaphragm fiber type based on myosin heavy chain isoforms

After determination of the force-pCa relationship, single fibers were stored in sample buffer at -80°C and, subsequently, myosin heavy chain isoforms were determined for each individual fiber using gel electrophoresis according to previously established methods [35]. Fibers were classified based on their myosin heavy chain isoforms as either Type IIA, Type IIX, Type IIX/IIB, Type IIB or slow.

### Contractile protein level determination and assessment of ROS mediated protein modifications by western blots

To determine if hyperglycemia induced alterations in the content of the contractile proteins, western blots of diaphragm homogenates were used to assess diaphragm levels of actin, actinin, tropomyosin and troponin T. In addition, since free radicals have been shown to modulate diaphragm dysfunction in a variety of animal models [23-25], we also examined diaphragm muscle homogenates for ROS-mediated protein modifications (nitrotyrosine side group formation, protein carbonyl formation). For these determinations, muscle samples were homogenized in buffer (10 mM beta-glycerophosphate, 50 mM sodium fluoride, 1 mM sodium, 20 mM 4-(2-hydroxyethyl)-1-piperazine-ethanesulfonic acid (HEPES), 2 mM ethylenediaminetetraacetic acid (EDTA), 250 mM sodium chloride, 2 microgram/ml leupeptin, 2 microgram/ml aprotinin, 1 mM PMSF, 0.5 microgram/ml benzamidine, and 1 mM dithiothreitol (DTT)) in a 1 gm/10 ml ratio, centrifuged at 3,000 g for 10 minutes. Protein contents of supernatants were assessed using the Bradford assay (BioRad Laboratories, Hercules, CA, USA). Supernatants were then diluted 1:1 with loading buffer (126 mM Tris–HCl, 20% glycerol, 4% SDS, 1.0% 2-mercaptoethanol, 0.005% bromophenol blue, pH 6.8), boiled for five to seven minutes and equal amounts of protein (2 to 10 micrograms) were loaded onto Tris-glycine polyacrylamide gels. Proteins were separated by electrophoresis (Novex Minicell II, Carlsbad, CA, USA), transferred to polyvinylidene fluoride (PDVF) membranes and incubated over night at 4°C with primary antibodies to targeted proteins. The following reagents were used: anti-actin, anti-actinin, anti-tropomyosin and anti-troponin T (Sigma Aldrich, St. Louis, MO, USA), and anti-nitrotyrosine (EMD Millipore Corporation, Billerica, MA, USA). Protein carbonyl determinations were performed using the OxyBlot™ Protein Oxidation Detection Kit (EMD Millipore Corporation) following the manufacturer’s instructions. Following incubation in the primary antibody, membranes were washed and then subsequently incubated with horseradish peroxidase (HRP)-conjugated secondary antibodies and antibody binding detected using enhanced chemiluminescence (Western Lightning®-ECL, Perkin Elmer, Waltham, MA, USA). Densitometry was performed using a Microtek scanner (Carson, CA, USA) and UN-SCAN-IT software (Silk Scientific, Orem, UT, USA). To verify equal loading of lanes, blots were stripped and reprobed with anti-tubulin (Sigma Aldrich).

### Statistical analysis

Analysis of variance (ANOVA) was employed to compare variables (for example, force) across groups of animals treated with different agents, with *post-hoc* testing (Tukey) to determine differences between groups. A *P* <0.05 was taken as indicating statistical significance. Data are presented as ± 1 standard error of the mean (SEM).

## Results

### Animal characteristics

Control animals were euglycemic with glucose levels averaging 105 + 4 mg/dl (Table 1). At the time of sacrifice, glucose levels were greater than 350 mg/dl in all three STZ-treated groups. Importantly, administration of either active PEG-SOD or denatured PEG-SOD had no effect to lower glucose levels. Total body weight decreased significantly in all hyperglycemia groups; the reduction in body mass averaged 11 ± 2%, 13 ± 2% and 15 ± 1% for hyperglycemia, hyperglycemia + PEG-SOD and hyperglycemia + denatured PEG-SOD groups, respectively (*P* <0.001), when compared to control animals in which total body weight increased by 13 ± 4%. Total costal diaphragm weights also were significantly reduced in all hyperglycemia groups when compared to controls (Table 1). To further examine the effects of hyperglycemia and/or administration of PEG-SOD on diaphragm atrophy, we assessed the ratio of final diaphragm weights to final animal weights, reasoning that reductions in this ratio would be indicative of preferential diaphragm atrophy, rather than generalized reductions in muscle mass related to the global reduction in total body mass. As shown in the figure in Additional file 1: Figure S1, there was a trend for reductions in this ratio for the HG and HG + denatured PEG-SOD treated groups compared to the control group and HG + PEG-SOD groups, but these comparisons were not statistically significant (*P* = 0.24).

**Table 1:** Animal data

**Experimental group**	**Initial animal weight (grams)**	**Final animal weight (grams)**	**Costal diaphragm weight (mg)**	**Glucose (mg/dL)**
Control	306 ± 13	342 ± 10	725 ± 61	105 ± 5
Hyperglycemia	295 ± 13	261 ± 9*	475 ± 31*	434 ± 18*
Hyperglycemia + PEG-SOD	325 ± 13	282 ± 9*	566 ± 26*	485 ± 15*
Hyperglycemia + denatured PEG-SOD	297 ± 9	253 ± 12*	453 ± 38*	489 ± 7*

*Significantly different when compared to control group (*P* <0.001).

### Effects of hyperglycemia on diaphragm specific force generation

**Figure 1:**
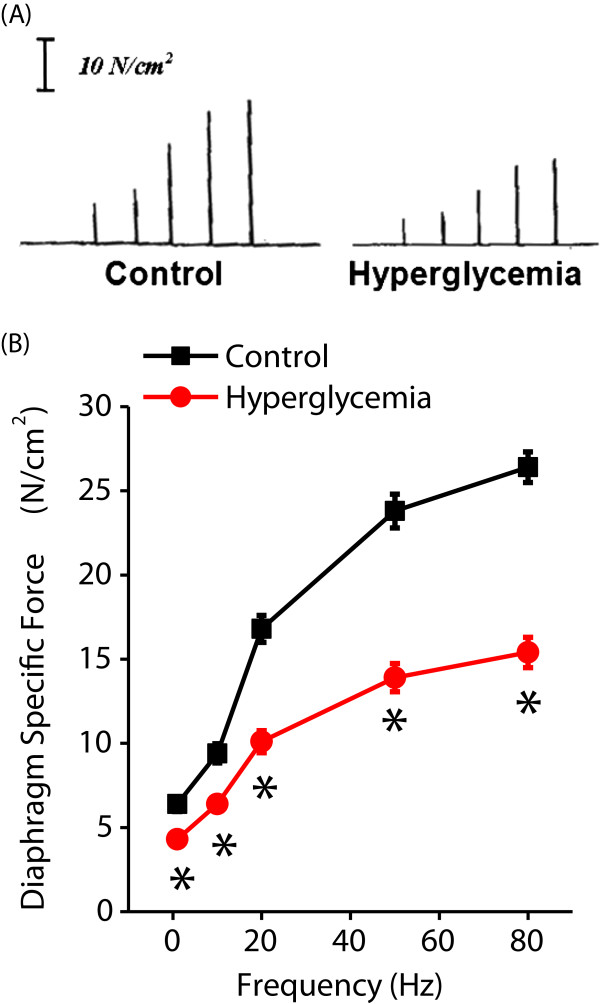
**Hyperglycemia alters diaphragm force-frequency curves. A)** demonstrates representative force frequency curves (raw data from 1, 10, 20, 50 and 80 Hz stimulation frequencies) generated for intact diaphragm strips from a control animal treated with citrate buffer alone (left) and from a hyperglycemic animal two weeks after the initial injection of STZ (right). **B)** demonstrates mean data ± SEM (n = 6) for control (black) and two week hyperglycemic animals (red). As shown, hyperglycemia induced large reductions in the diaphragm specific force generation (force/cross sectional area) at all stimulation frequencies when compared to control animals. (*P = .002 for 1 Hz, *P* ≤0.001 for all other stimulation frequencies). SEM, standard error of the mean; STZ, streptozotocin.

The total force generated by a muscle is the product of muscle size (that is, total cross sectional area) and the force generating capacity per unit cross sectional area, otherwise known as muscle specific force. As shown in Figure 1, hyperglycemia resulted in a large reduction in diaphragm specific force, decreasing levels of force generated across the entire range of stimulation frequencies tested (*P* = .002 for 1 Hz, *P* <0.001 for all others). These data confirm the previous report by Hida *et al*. which showed that diaphragm specific force generation is reduced after two weeks of hyperglycemia [27].

### Effects of hyperglycemia on diaphragm single fiber force-pCa relationships

**Figure 2:**
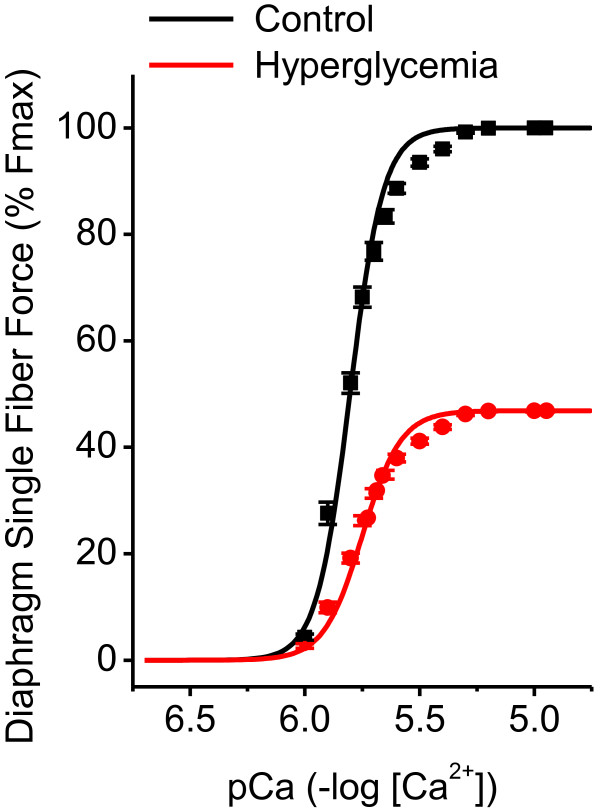
**Hyperglycemia alters the force-pCa relationship in single permeabilized diaphragm fibers.** Average force-Ca curves were constructed for single fibers from control (black) and two week hyperglycemic animals (red) using the Hill equation (15 fibers from each of six animals per condition for a total of 90 fibers per group). Symbols and error bars represent the mean ± SEM of the data points obtained for each individual fiber. Force (Fmax) is the absolute force generation per cross sectional area of each individual fiber normalized to the percentage of the control fibers. pCa represents the (-log [Ca ^2+^]) indicating that the calcium content in the solution increases along the X-axis. As shown, two weeks of hyperglycemia significantly alters the force-pCa relationship in single permeabilized diaphragm fibers, indicating that most of the hyperglycemia-induced diaphragm force reductions are due to alterations at the level of the contractile proteins (*P* <0.001 for force in fibers from hyperglycemic groups compared to control fibers at all pCa values greater than 6.0). SEM, standard error of the mean.

To determine if the loss of diaphragm force generation in response to hyperglycemia was the consequence of alterations at the level of the contractile proteins, we assessed the force-pCa relationship in 360 single permeabilized diaphragm fibers (Figure 2). We found that hyperglycemia induced a large downshift in the diaphragm contractile protein force-pCa relationship, reducing Fmax from 182.9 ± 1.85 kPa to 85.7 ± 1.99 kPa (this is an index of actin-myosin crossbridge formation) (*P* <0.001) and decreasing the coefficient of cooperativity, N, from 5.98 to 5.17 (*P* = 0.015). Hyperglycemia did not, however, alter the calcium sensitivity (pCa_50_) of the contractile proteins (Table 2).

**Table 2:** Data from single permeabilized diaphragm fibers

	**Experimental group**	**Maximum force (kPa)**	**N value**	**pCa**_**50**_
Control	182.9 ± 1.8	5.98 ± 0.19	5.81 ± 0.01
Hyperglycemia	85.7 ± 2.0*	5.17 ± 0.27*	5.69 ± 0.07
Hyperglycemia + PEG-SOD	148.6 ± 2.4*	5.76 ± 0.22	5.78 ± 0.01
Hyperglycemia + Denatured PEG-SOD	90.9 ± 1.5*	4.33 ± 0.18*	5.72 ± 0.01

Single fiber maximum force is reported as absolute force generation per fiber cross sectional area in kPa. N is the Hill coefficient and indicates thin filament cooperativity. pCa50 is the calcium concentration at which half maximal activation occurs. Data are presented as the mean ± standard error of the mean (SEM) and include all fiber types for each experimental group (n = 90/group). *Significantly different when compared to control group (*P* < 0.001).

### Effect of PEG-SOD on diaphragm specific force generation in intact muscle strips

**Figure 3:**
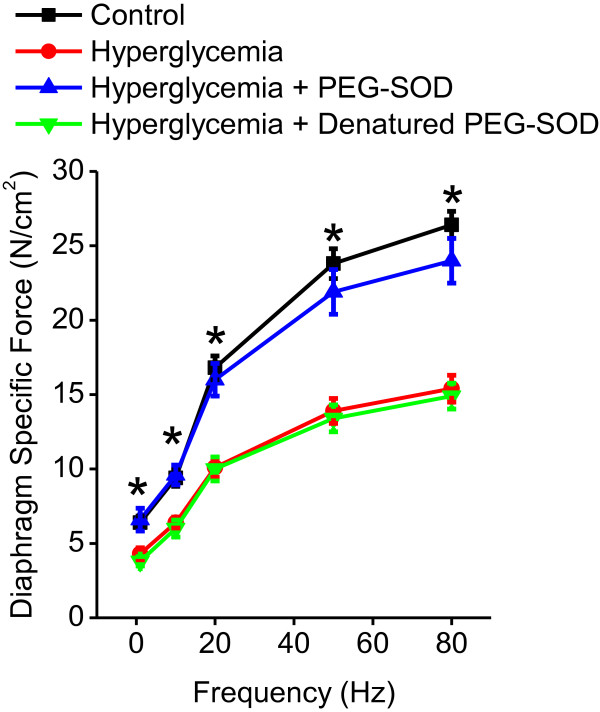
**Polyethylene glycol superoxide dismutase (PEG-SOD) restores hyperglycemia-induced reductions in the diaphragm force-frequency response.** Data are shown for intact diaphragm strips from control (black), hyperglycemia (red), hyperglycemia + PEG-SOD (blue) and hyperglycemia + denatured (heat inactivated) PEG-SOD groups (green) obtained at two weeks after administration of citrate buffer (controls) or streptozotocin (hyperglycemia) (n = 6 animals/group). PEG-SOD and denatured PEG-SOD were administered intraperitoneally at Day 7 after the initial injection for a total duration of seven days. Administration of PEG-SOD largely restored diaphragm force generation in hyperglycemic animals to that of controls, whereas denatured PEG-SOD had no effect; recovery of diaphragm specific force with PEG-SOD was not due to normalization of glucose levels. (*P* ≤0.001, *control and hyperglycemic + PEG-SOD groups significantly different compared to hyperglycemia and hyperglycemia + denatured PEG-SOD groups).

Administration of PEG-SOD largely reversed the effects of hyperglycemia on diaphragm specific force generation, restoring the force-frequency relationship measured on intact diaphragm strips (Figure 3). Specifically, diaphragm specific force generation (for example, 80 Hz force) was 26.4 ± 0.9, 15.4 ± 0.9, 24.0 ± 1.5 and 14.9 ± 0.9 N/cm^2^ for control, two week hyperglycemia, two week hyperglycemia + PEG-SOD and two week hyperglycemia + denatured PEG-SOD groups, respectively (*P* <0.001). As shown in Figure 3, PEG-SOD restored force generation at all frequencies tested, similar to levels seen in control euglycemic animals.

### Effect of PEG-SOD on diaphragm single fiber force-pCa relationships

**Figure 4:**
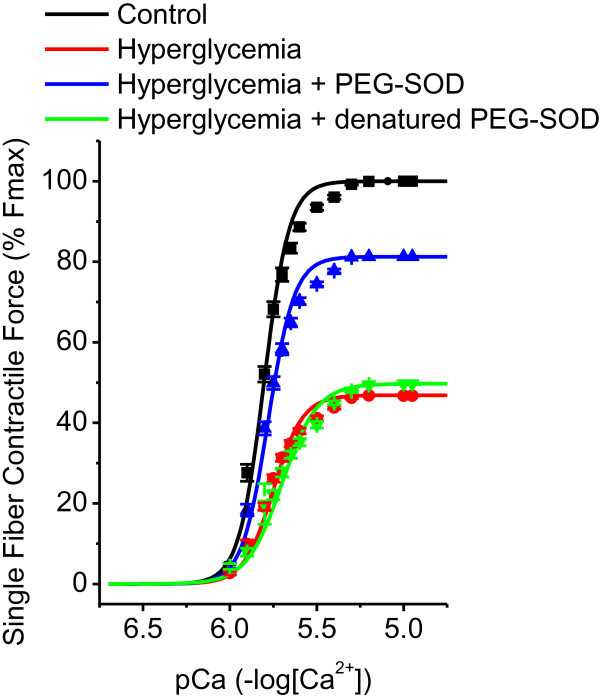
**PEG-SOD restores hyperglycemia-induced reductions in diaphragm permeabilized single fiber contractile force generation.** Force pCa relationships in single permeabilized diaphragm fibers from control (black), hyperglycemia (red), hyperglycemia + PEG-SOD (blue), and hyperglycemia + denatured (heat inactivated) PEG-SOD groups (green) were evaluated at two weeks after administration of citrate buffer (controls) or streptozotocin (hyperglycemia) (n = 6 animals/group). A total of 15 fibers from each animal were assessed (90 fibers/experimental group, total 360 fibers). As shown, PEG-SOD substantially improved the force-pCa relationship in single permeabilized diaphragm fibers from hyperglycemic animals, whereas denatured PEG-SOD had no effect to restore single fiber force generating capacity (*P* < 0.001). PEG-SOD did not normalize glucose levels. These data suggest that inhibition of superoxide generation in the hyperglycemic diaphragm restores contractile protein function. PEG-SOD, polyethylene glycol superoxide dismutase.

We also assessed single diaphragm fiber force-pCa relationships in hyperglycemic animals given PEG-SOD and dnPEG-SOD. We found that PEG-SOD restored Fmax and N values to control levels and shifted force-pCa curves to levels similar to those for fibers from control, euglycemic animals (Figure 4) (*P* <0.001 for Fmax and *P* <0.001 for N values, respectively).

**Figure 5:**
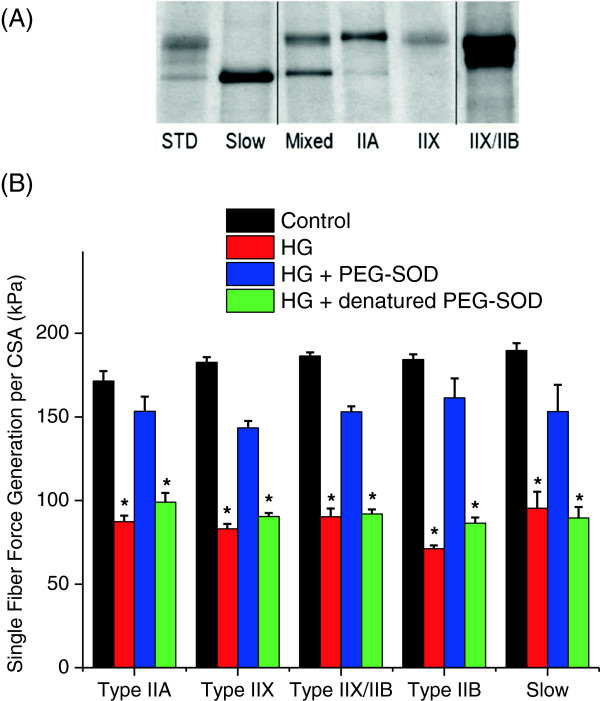
**Hyperglycemia reduces single fiber contractile force generation in all fiber types.** Fiber typing was performed for each diaphragm fiber in which force-pCa relationships were determined and then evaluated to assess if the changes in force were related to a specific fiber type. A total of 346 fibers were typed and subsequently classified as Type I (slow), Type IIA, Type II B, Type IIX, Type IIB/X and mixed based on the migration pattern of myosin heavy chains (determinations were not made for 14 fibers due to technical issues). **A)** depicts a representative gel for determination of individual fiber type. The first lane is a protein standard obtained from total diaphragm homogenates which contains all myosin heavy chain isoforms, the other lanes indicate myosin heavy chain isoforms from individual fibers. The image was obtained from the same gel, but lanes were not adjacent and are demarcated by the lines within the representative image. **B)** represents the effect of fiber type on maximal single fiber contractile force generation in response to hyperglycemia, hyperglycemia + PEG-SOD and hyperglycemia + denatured (heat inactivated) PEG-SOD. The absolute force generation/CSA in kPa is indicated for the different fiber types from the four experimental groups. As shown, hyperglycemia reduced single fiber contractile force generation in all diaphragm fiber types. Administration of PEG-SOD, but not denatured PEG-SOD, largely restored contractile force generation in single permeabilized diaphragm fibers independent of fiber type (*P* < 0.001, * significantly different when compared to control and hyperglycemia + PEG-SOD fibers). PEG-SOD, polyethylene glycol superoxide dismutase.

Previous studies which have examined force-pCa relationships in the rat diaphragm have reported fiber type specific changes in contractile protein characteristics [35,36] as well as fiber type specific atrophy in a variety of animal models (chronic obstructive pulmonary disease (COPD), chronic corticosteroid treatment, cervical spinal cord injury induced inactivity and mechanical ventilation) [37]. To determine if hyperglycemia induced alterations in single fiber force-pCa relationships were fiber-type specific, we analyzed single fiber maximal force generation, pCa 50, N values (obtained from the Hill equation), and fiber cross sectional area for Type IIA, IIX, IIX/IIB, IIB and slow fibers across the experimental groups (see the table in Additional file 2: Table S1 for details of this analysis). We found that the protective effect of PEG-SOD on force generation in single fibers was observed in all diaphragm fiber types (Figure 5). However, there were no fiber type specific differences in pCa50 values (calcium sensitivity) or N values (Hill coefficient) between control, HG, HG + PEG-SOD and HG + dnPEG-SOD groups. Hyperglycemia significantly decreased Type IIA fiber cross sectional area, which was not restored by administration of PEG-SOD.

### Contractile protein levels and indices of protein oxidation

There are several mechanisms by which pathological stresses can alter contractile protein function. One possible mechanism is via activation of proteolytic pathways with resultant cleavage and loss of specific contractile elements. A second process is via chemical modification of contractile elements through kinase-mediated phosphorylation reactions or sidegroup modifications by reactive species (for example, nitrosylation of tyrosine residues by peroxynitrite or carbonyl formation in response to reaction with ROS species). Moreover, a number of previous studies have shown that oxidative stress in the diaphragm is associated with alterations in diaphragm contractile performance [26,38,39].

**Figure 6:**
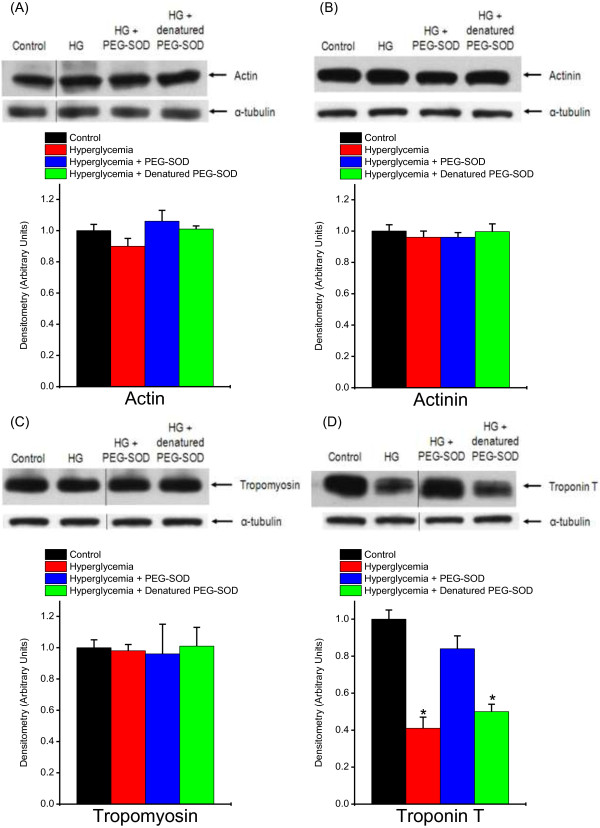
**Hyperglycemia induces selective depletion of diaphragm troponin T.** Representative western blots and mean densitometry of key contractile proteins from diaphragm homogenates from control (black), hyperglycemic (red), hyperglycemic + PEG-SOD (blue), and hyperglycemic + denatured (heat inactivated) PEG-SOD groups (n = 6 for each comparison). Images were obtained from the same gel, but lanes were not adjacent and are demarcated by the lines within the representative image. Actin levels are shown in **A)**, actinin levels in **B)**, tropomyosin levels in **C)** and troponin T levels in **D)**. Blots were reprobed with tubulin as a loading control. Densitometry is presented in arbitrary units and was normalized to control values. There were no significant differences in the protein contents of actin, actinin, or tropomyosin between groups. On the other hand, troponin T levels were significantly decreased with hyperglycemia; this loss of troponin T was prevented with administration of PEG-SOD, but not with denatured PEG-SOD (*P* <0.001). These data demonstrate that a superoxide scavenger preserves hyperglycemia -induced loss of troponin T in the diaphragm. (* significantly different from control and hyperglycemic + PEG-SOD groups). PEG-SOD, polyethylene glycol superoxide dismutase.

To determine if hyperglycemia altered the level of contractile proteins, we measured levels of four proteins known to play key functions in contractile force generation, including actin, actinin, tropomyosin and troponin T (Figure 6). We found that hyperglycemia did not result in depletion of either actin, actinin or tropomyosin but significantly reduced levels of troponin T, one of the key proteins involved in the regulation of crossbridge cycling. We also found that PEG-SOD attenuated this selective loss of troponin T, restoring levels to near control values (*P* = 0.001 for comparison of all groups).

**Figure 7:**
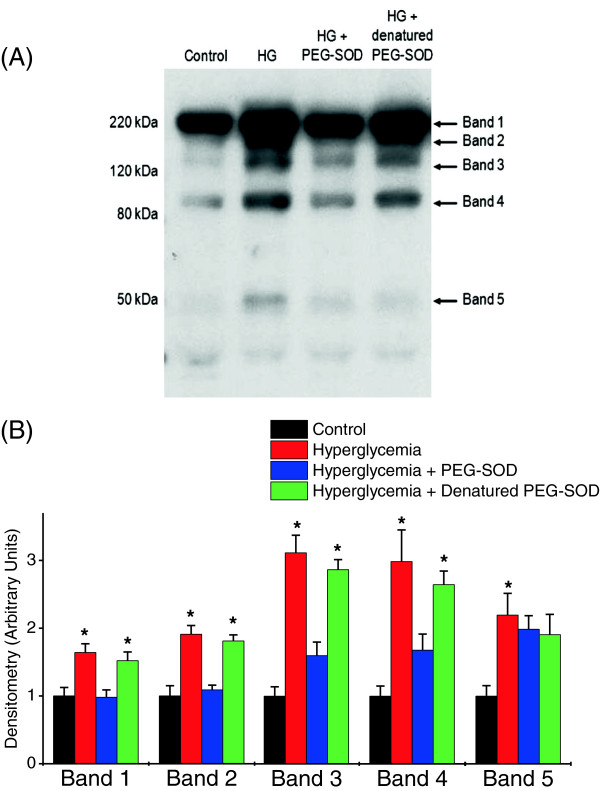
**Hyperglycemia increases tyrosine nitration of several diaphragm proteins. A)** is a representative blot for nitrotyrosine staining in diaphragm homogenates from control (black), hyperglycemic (red), hyperglycemic + PEG-SOD (blue), and hyperglycemic + denatured (heat inactivated) PEG-SOD animals. **B)** indicates mean ± SEM of the densitometric analyses for Bands 1 to 4 (n = 6 for comparisons between the four experimental groups). As shown, at least four band differences were noted between control and hyperglycemic diaphragm homogenates; administration of PEG-SOD to hyperglycemic animals largely reversed the hyperglycemia-induced changes in nitrotyrosine staining in diaphragm homogenates, suggesting that the PEG-SOD-induced reductions in superoxide generation prevented peroxynitrite formation and subsequent tyrosine nitration of proteins. These effects were not seen with administration of denatured PEG-SOD. (*P* <0.001, *significantly different when compared to control or hyperglycemia + PEG-SOD). PEG-SOD, polyethylene glycol superoxide dismutase; SEM, standard error of the mean.

**Figure 8:**
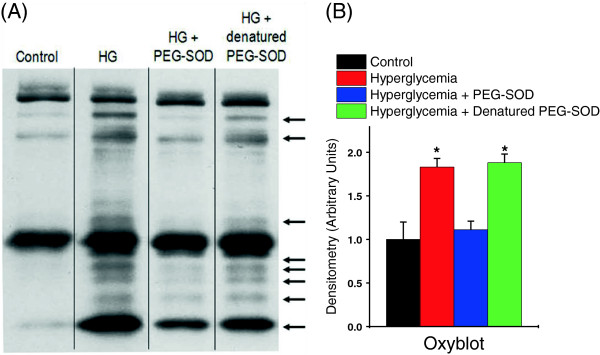
**Hyperglycemia increases protein carbonyl modifications in the diaphragm. A)** is a representative oxyblot in diaphragm homogenates from the four experimental groups. Image was obtained from the same gel, but lanes were not adjacent and are demarcated by the lines within the representative image. As shown, hyperglycemia produced multiple protein carbonyl modifications of diaphragm proteins. **B)** is the mean ± SEM of the total lane densitometry of the four experimental groups (n = 4). Administration of PEG-SOD, but not denatured PED-SOD, largely abolished these modifications indicating that excessive superoxide generation mediates this process. (*P* <0.001, *significantly different when compared to control or hyperglycemia + PEG-SOD). PEG-SOD, polyethylene glycol superoxide dismutase; SEM, standard error of the mean.

We also assessed if hyperglycemia induced protein modifications in the diaphragm known to be associated with oxidative stress (that is, nitrotyrosine and protein carbonyl formation). As shown in Figure 7, hyperglycemia induced large increases in diaphragm proteins with nitrosylated side groups, including a protein migrating with a molecular weight characteristic of myosin (Band 1). In addition, hyperglycemia significantly increased diaphragm protein carbonyl side group content (Figure 8) (*P* <0.001), with large increases in the carbonyl content of nine protein bands. Administration of PEG-SOD largely ablated all hyperglycemia -induced increases in nitrotyrosine and protein carbonyl side group formation, suggesting that these increases are produced by ROS-mediated mechanisms. Administration of inactive, denatured PEG-SOD did not prevent hyperglycemia-induced increases in either diaphragm nitrotyrosine or protein carbonyl content.

## Discussion

While a limited number of studies have examined the effects of hyperglycemia on the diaphragm, the majority of these reports have assessed changes in diaphragm force generation at much longer durations of hyperglycemia (four to eight weeks) compared to the current study, with some studies reporting increases in diaphragm specific force, while others report decreases [40,41]. However, neither of these studies investigated the role of ROS in the diaphragm alterations they observed. We are aware of only one previous study that assessed diaphragm specific force generation after two weeks of hyperglycemia, reporting reductions in diaphragm specific force generation similar to those that we observed and suggesting a possible link between ROS mediated processes and reductions in diaphragm contractility [27]. However, this study did not examine intracellular targets of hyperglycemia-induced changes in the diaphragm, did not examine single diaphragm fiber force generation, did not assess contractile protein content and did not measure any indices of diaphragm oxidative stress in response to hyperglycemia.

In contrast to this previous work, we found that hyperglycemia induced reductions in diaphragm contractility occur largely at the level of the contractile proteins with resultant loss of diaphragm troponin T and increased indices of oxidative stress. Moreover, the present work is the first to show that administration of a selective superoxide scavenger, PEG-SOD, completely reversed hyperglycemia-induced diaphragm weakness, increasing force generation in response to the entire range of physiologically relevant stimulation frequencies. In addition, by using single permeabilized fiber techniques, we demonstrate that the contractile proteins are a major intracellular target of hyperglycemia with hyperglycemia inducing both a reduction in maximum contractile protein force generation and a reduction in thin filament interactions. We also show that administration of PEG-SOD reverses hyperglycemia -induced alterations in contractile protein function, improving maximum force generating capacity and restoring thin filament cooperativity, providing evidence that this process is, in part, mediated by free radical generation and, specifically, by excessive superoxide generation.

It is known that hyperglycemia increases free radical generation in tissues other than skeletal muscle [17,18,42]. More importantly, this previous work also indicates that excessive generation of superoxide and other ROS (for example, peroxynitrite, hydrogen peroxide) plays an important role in the genesis of hyperglycemia-induced tissue damage in several organs [17,18,43,44]. For example, hyperglycemia-induced endothelial dysfunction is thought to be linked to excessive glucose entry into endothelial cells leading to an increase in NADH production within the mitochondria, enhanced reduction of the proximal portion of the electron transport chain and resultant increases in electron transport chain superoxide generation [45,46]. Inhibition of this mechanism of superoxide generation has been shown to prevent hyperglycemia-induced endothelial dysfunction in animal models [32]. Similarly, hyperglycemia-induced retinal damage has been linked to retinal superoxide generation by NADPH oxidase, with reports indicating that administration of superoxide scavengers prevents diabetes-induced retinal damage [17,18]. The present study extends this previous work, indicating that a superoxide scavenger can also prevent hyperglycemia-induced diaphragm weakness. We did not identify the source of excessive diaphragm ROS generation in response to hyperglycemia in the present study, but these previous studies as well as studies examining ROS production in skeletal muscle, suggest a number of potential sites including the mitochondrial electron transport chain, NADPH oxidase, xanthine oxidase and uncoupled nitric oxide synthase [47,48]. While mitochondria are implicated as the most important source of superoxide in skeletal muscle, a recent study suggests that NADPH oxidase is the major source of superoxide generation in skeletal muscle at rest and during contraction [49]. Ongoing studies in our lab are designed to elucidate which of these sources of superoxide generation are pathogenetically linked to the development of hyperglycemia-induced diaphragm dysfunction.

Our observation that ROS play a role in producing diaphragm weakness during hyperglycemia is consistent with several previous reports indicating that excessive ROS generation mediates the development of diaphragm dysfunction in a variety of animal models of disease including congestive heart failure, mechanical ventilation-induced inactivity, sepsis, and during fatigue [23,25,50,51]. In these other conditions, as in the present study, there is evidence of excessive ROS generation in the diaphragm, protection from force loss when superoxide scavengers are administered, and superoxide dependent reductions in parameters of single fiber contractile protein function (for example, reductions in the Fmax) [24]. Additional data suggest that there are several mechanisms by which free radical species alter contractile protein function. First, we have shown that when various free radical generating solutions are applied directly to the diaphragm single fiber contractile proteins, superoxide anions, hydroxyl radicals and peroxynitrite each depress maximal contractile protein force generation (Fmax) [32,34]. In addition to these direct effects on contractile proteins, ROS have been shown to activate a variety of signaling kinases in skeletal muscle including JNK, PKR and p38 [52-54]. These kinases, in turn, trigger alterations in a variety of downstream pathways in skeletal muscle, including the intrinsic caspase pathway, the extrinsic caspase pathway, components of the proteasomal degradation pathway and factors regulating protein translation (for example, eIF2α, SK6) [29,30,55-62]. In addition, ROS are also known to activate the calpain proteolytic system in skeletal muscle [58,61]. While the exact effects of activated calpain, the activated proteasomal system and a sudden reduction in protein translation on contractile protein function are not known, activated caspase induces alterations in contractile protein function that are similar to those observed in response to hyperglycemia in the present study, that is, caspase induces large reductions in Fmax and alters N, the index of thin filament cooperativity [63]. In addition, Smuder *et al*. reported that oxidative modification of diaphragm myofibrillar proteins increases their susceptibility to caspase and calpain mediated degradation [57]. As a result, the pattern of alterations in contractile protein function induced by hyperglycemia in the present study is similar to that seen in response to several known effects of ROS, that is, the direct effects of ROS on contractile protein function and the effects of ROS triggered proteolysis on the contractile proteins. We also found that hyperglycemia induced a significant reduction in troponin T levels, with this effect inhibited by PEG-SOD administration. Troponin T is a target of both activated caspase and activated calpain [64,65], and it is possible that hyperglycemia induced loss of this particular contractile protein through ROS mediated activation of these proteolytic enzymes. Additional work will be needed to determine the exact role of the various downstream effects of ROS in altering contractile protein function in hyperglycemia and to also evaluate the specific mechanisms responsible for the observed depletion of troponin.

### Limitations

There are several elements of our methodology that require careful consideration. We chose to use the STZ model to induce hyperglycemia in our animals. While this is a well established model of hyperglycemia which reproducibly produces similar, sustained elevations in blood glucose levels in animals, a potential criticism is that STZ *per se* could have some unrecognized toxicity that may directly affect muscle function, potentially influencing our results. To exclude this possibility, we administered insulin to STZ animals (n = 3) and compared the force-frequency relationship in insulin-treated STZ animals to those animals given STZ alone, reasoning that if STZ *per se* was responsible for the alterations in diaphragm function we observed, these abnormalities would not improve with restoration of euglycemia. We found that insulin treatment markedly improved diaphragm contractility in STZ-treated animals (see figure in Additional file 3: Figure S2), indicating that in our model, diaphragm weakness is not due to a toxic effect of STZ, but rather, is secondary to the effects of hyperglycemia.

Another potential criticism is that the STZ model does not completely replicate the hyperglycemia of stress seen in critically ill patients. Studies have suggested that hyperglycemia in ICU patients primarily arises from increased insulin resistance rather than loss of insulin secretion [66]. In the present study, we employed a dose of STZ that was sufficiently low such that complete loss of insulin secretion would not occur [67,68]. Moreover, at these doses of STZ, animals gradually develop peripheral insulin resistance over a period of weeks [69]. As a result, this model induces a form of hyperglycemia (peripheral resistance to insulin, moderate reductions in insulin levels) that is a reasonable simulation of what occurs in critically ill patients. Recently, other investigators have used animal models of critical illness (trauma, hemorrhage, sepsis, burn injury) in combination with glucose loading to mimic stress induced hyperglycemia [70-72]. However, use of such complex models would make it difficult to discriminate between the effects of the underlying injury from the effects of hyperglycemia on diaphragm function. Nevertheless, it will be of interest to determine if the same phenomena observed in the present study are also observed in some of these other animal models.

Our data provide strong evidence that hyperglycemia-induced superoxide generation mediates diaphragm weakness. However, in the present study, we did not identify the intracellular source of superoxide. As indicated above, we are performing additional studies to identify the precise source of superoxide generation in the diaphragm in response to hyperglycemia. In addition, the fact that we observed increased nitrotyrosine formation in diaphragm homogenates suggests that hyperglycemia may also result in excessive generation of nitric oxide and/or reactive nitrogen species, and the role of nitric oxide in the genesis of hyperglycemia-induced diaphragm weakness warrants further exploration. Finally, while we provide evidence of ROS mediated protein modifications in the diaphragm, in addition to depletion of troponin T, future studies will be needed to identify further other protein modifications as well as the proteolytic processes that may account for hyperglycemia-induced physiologic derangements in diaphragm contractility. Nonetheless, our findings represent an important first step in characterizing the effects of hyperglycemia on respiratory muscle function.

## Conclusions

Previous work indicates that poor glycemic control in critically ill patients increases the incidence of ICU acquired diaphragm weakness and prolongs duration of mechanical ventilation. The present study provides mechanistic information regarding the effects of hyperglycemia on diaphragm contractility, and potentially explains how this specific risk factor might contribute to diaphragm weakness and impact weaning from mechanical ventilation in critically ill patients. The obvious treatment for hyperglycemia in critical illness is insulin therapy; however, the data that indicate that this approach prevents ICU acquired diaphragm weakness show that strict glycemic control with intensive insulin therapy is required. Moreover, recent studies suggest that this approach increases ICU mortality, primarily due to complications arising from hypoglycemia [73-75], and as such, current clinical recommendations for the management of hyperglycemia in critically ill patients do not support the use of strict glucose control. Importantly, the data from the present study show that hyperglycemia-induced respiratory muscle weakness can be prevented by administration of a superoxide scavenger (PEG-SOD), as this agent dramatically restored diaphragm function in hyperglycemic animals despite having no effect on glucose levels. Moreover, diaphragm weakness improved dramatically even though treatment with PEG-SOD was delayed for one week after hyperglycemia was present. Therefore, it seems reasonable to speculate that treatment with agents that target ROS may provide an alternative approach to prevent hyperglycemia-induced respiratory muscle dysfunction in critical illness. Nonetheless, additional clinical studies will be needed to assess the efficacy of such therapies in mechanically ventilated hyperglycemic ICU patients.

## Key messages

• Hyperglycemia induces diaphragm weakness and is associated with loss of troponin T and increased markers of oxidative stress.

• Alterations in diaphragm specific force generation in response to hyperglycemia occur at the level of the contractile proteins and are independent of diaphragm fiber type.

• Administration of PEG-SOD, a specific scavenger of superoxide, prevents hyperglycemia-induced diaphragm weakness, restoring both intact diaphragm force generation as well as single fiber diaphragm force generation without lowering glucose levels.

• Our data identify new mechanisms for hyperglycemia induced diaphragm weakness, and potentially explain how poor glucose control might potentiate the development of diaphragm weakness and prolong the duration of mechanical ventilation during critical illness.

## Abbreviations

ANOVA: analysis of variance; Ca50: calcium concentration required for half-maximal activation; COPD: chronic obstructive pulmonary disease; CSA: cross sectional area; dnPEG-SOD: denatured polyethylene glycol conjugated superoxide dismutase; DTT: dithiothreitol; EGTA: ethylene glycol tetraacetic acid; HEPES: 4-(2-hydroxyethyl)-1-piperazine-ethanesulfonic acid; HG: hyperglycemia; HRP: horseradish peroxidase; ICUAW: intensive care unit acquired weakness; JNK: c-Jun N-terminal kinase; kPa: kilopascal; NAC: N-acetyl cysteine; NADH: reduced form of nicotinamide adenine dinucleotide; NADPH oxidase: nicotinamide adenine dinucleotide phosphate-oxidase; pCa: -[log] calcium concentration; PDVF: polyvinylidene fluoride; PEG-SOD: polyethylene glycol conjugated superoxide dismutase; PKR: double-stranded RNA-dependent protein kinase; PMSF: phenylmethanesulfonylfluoride; ROS: reactive oxygen species; SDS: sodium dodecyl sulfate; SEM: standard error of the mean; STZ: streptozotocin.

## Competing interests

Both authors declare that they have no competing interests.

## Authors’ contributions

LAC and GSS conceived and designed the study, supervised the research process, analyzed and interpreted the data, drafted the manuscript and approved the final manuscript. Both authors read and approved the final manuscript.

## Supplementary Material

Additional file 1: Figure S1 Final diaphragm weight to final animal weight ratios. This is a graph demonstrating the final diaphragm weight to final animal weight ratios in the experimental groups. Click here for file

Additional file 2: Table S1 Fiber type specific data in single permeabilized diaphragm fibers from all experimental groups. This is a table showing the detailed analyses of single fiber experiments based on fiber type as determined by the myosin heavy chain content in individual fibers; included are fiber type specific parameters of maximal force generation per cross sectional area (kPa), cross sectional area, N values (Hill coefficient) and the pCa_50_. Click here for file

Additional file 3: Figure S2 Effect of insulin treatment on diaphragm specific force generation. This figure demonstrates the effects of insulin treatment in hyperglycemic animals on the diaphragm specific force generation. Click here for file
