# Supplementary material for: Hyperglycemia-induced diaphragm weakness is mediated by oxidative stress
Source: Crit Care. 2014 May 3;18(3):R88. doi: 10.1186/cc13855 (PMC4056378; doi:10.1186/cc13855)
Supplement: Additional file 1: Figure S1 — Final diaphragm weight to final animal weight ratios. This is a graph demonstrating the final diaphragm weight to final animal weight ratios in the experimental groups. [file cc13855-S1.docx]

Additional file 1: Figure S1. Final Diaphragm Weight to Final Animal Weight Ratios

We assessed the ratio of costal diaphragm weights (mg) to final animal weights (gms) to determine if there was preferential atrophy of the diaphragm in response to hyperglycemia. While this ratio for the HG (red) and HG + denatured PEG-SOD (green) treated groups tended to be lower compared to the control group (black) and HG+PEG-SOD (blue) groups, these comparisons were not statistically significant (p=0.24).
