# Supplementary material for: Hyperglycemia-induced diaphragm weakness is mediated by oxidative stress
Source: Crit Care. 2014 May 3;18(3):R88. doi: 10.1186/cc13855 (PMC4056378; doi:10.1186/cc13855)
Supplement: Additional file 2: Table S1 — Fiber type specific data in single permeabilized diaphragm fibers from all experimental groups. This is a table showing the detailed analyses of single fiber experiments based on fiber type as determined by the myosin heavy chain content in individual fibers; included are fiber type specific parameters of maximal force generation per cross sectional area (kPa), cross sectional area, N values (Hill coefficient) and the pCa50. [file cc13855-S2.docx]

Additional file 2: Table S1. Fiber Type Specific Data in Single Permeabilized Diaphragm Fibers from All Experimental Groups

| **MHC**  **Fiber Type** | **Parameters** | **Control** | **HG** | **HG+PEG-SOD** | **HG+dnPEG-SOD** |
| --- | --- | --- | --- | --- | --- |
| **Type IIA** |  |  |  |  |  |
|  | Force (kPa) | 171± 6 | 87 ± 4* | 171 ± 6 | 99 ± 5* |
|  | CSA (µm^2^) | 2836 ± 622^†^ | 1038 ± 301 | 1416 ± 336 | 798 ± 259 |
|  | N | 5.8 ± 0.6 | 4.1 ± 0.4 | 6.2 ± 0.8 | 3.5 ± 0.7 |
|  | pCa50 | 5.81 ± 0.03 | 5.78 ± 0.03 | 5.80 ± 0.03 | 5.79 ± 0.08 |
| **Type IIX** |  |  |  |  |  |
|  | Force (kPa) | 183 ± 3 | 83 ± 3* | 144 ± 4 | 90 ± 2* |
|  | CSA (µm^2^) | 2389 ± 220 | 2353 ± 235 | 1959 ± 176 | 1539 ± 159 |
|  | N | 6.3±0.3 | 5.2 ± 0.3 | 5.8 ± 0.3 | 4.3 ± 0.2 |
|  | pCa50 | 5.81 ± 0.02 | 5.76 ± 0.10 | 5.79 ± 0.01 | 5.69 ± 0.02 |
| **Type IIX/IIB** |  |  |  |  |  |
|  | Force (kPa) | 186 ± 2 | 90 ± 5* | 153 ± 3 | 92 ± 3* |
|  | CSA (µm^2^) | 2630 ± 224 | 2662 ± 428 | 2128 ± 217 | 2059 ± 275 |
|  | N | 6.0 ± 0.3 | 6.8 ± 0.8 | 5.3 ± 0.4 | 4.5 ± 0.4 |
|  | pCa50 | 5.83 ± 0.01 | 5.81 ± 0.02 | 5.81 ± 0.01 | 5.76 ± 0.04 |
| **Type IIB** |  |  |  |  |  |
|  | Force (kPa) | 184 ± 3 | 71 ± 2* | 161 ± 11 | 86 ± 3* |
|  | CSA (µm^2^) | 2703 ± 150 | 1265 ± 276 | 2102 ± 635 | 1284 ± 372 |
|  | N | 5.7 ± 0.1 | 5.7 ± 0.2 | 5.1 ± 0.9 | 4.7 ± 1.0 |
|  | pCa50 | 5.79 ± 0.01 | 5.83 ±0.01 | 5.83 ± 0.03 | 5.78 ± 0.04 |
| **Slow** |  |  |  |  |  |
|  | Force (kPa) | 190 ± 4 | 95 ± 10* | 153 ± 16 | 89 ± 7* |
|  | CSA (µm^2^) | 1282 ± 241 | 1245 ± 546 | 1208 ± 130 | 892 ± 200 |
|  | N | 4.0 ± 0.6 | 3.1 ± 0.5 | 5.9 ± 1.4 | 3.9 ± 0.5 |
|  | pCa50 | 5.75 ± 0.03 | 5.72 ± 0.05 | 5.81 ± 0.05 | 5.70 ± 0.05 |

Single fiber maximum force is reported as absolute force generation per fiber cross sectional area in kPa. N is the Hill coefficient and indicates thin filament cooperativity. pCa50 is the calcium concentration at which half maximal activation occurs. Data are presented as the mean ± SEM. * significantly different when compared to control and HG+PEG-SOD group; ^†^ indicates significantly different from HG, HG+PEG-SOD, and HG+dnPEG-SOD groups.
