# Supplementary material for: Hyperglycemia-induced diaphragm weakness is mediated by oxidative stress
Source: Crit Care. 2014 May 3;18(3):R88. doi: 10.1186/cc13855 (PMC4056378; doi:10.1186/cc13855)
Supplement: Additional file 3: Figure S2 — Effect of insulin treatment on diaphragm specific force generation. This figure demonstrates the effects of insulin treatment in hyperglycemic animals on the diaphragm specific force generation. [file cc13855-S3.docx]

Additional file 3: Figure S2. Effect of Insulin Treatment on Diaphragm Specific Force Generation

Insulin (subcutaneously implanted sustained release pellets from LinShin Canada, Inc., Toronto, ON, CA) was administered to STZ-treated rats for two weeks following the initial injection of STZ to maintain glucose levels in the euglycemic range. As shown, treatment with insulin preserved the diaphragm force frequency relationship in STZ-treated animals, indicating that the reduction in diaphragm specific force generation observed in response to STZ was not due to STZ toxicity per se, but rather due to the effects of hyperglycemia.
